# Understanding the Impact of Mild Episodic Memory Difficulties on Everyday Tasks in Older Adults

**DOI:** 10.1002/alz.092933

**Published:** 2025-01-03

**Authors:** Molly B. Tassoni, Una Cutrone, Stephanie M. Simone, Sophia L. Holmqvist, Moira Mckniff, Kimberly Halberstadter, Marina Kaplan, Riya Chaturvedi, Melissa Rosahl, Anna Callahan, Tania Giovannetti

**Affiliations:** ^1^ Temple University, Philadelphia, PA USA; ^2^ Penn Alzheimer’s Disease Research Center, University of Pennsylvania, Philadelphia, PA USA

## Abstract

**Background:**

The Goal‐Control Model posits that episodic memory impairment leads to premature decay of everyday task goals, which contributes to task omissions (failure to accomplish task steps) in those with moderate to severe impairment. Although task omissions are not observed in those with mild episodic memory (mildEM) impairment, it has yet to be investigated if goal decay is reflected by subtle errors during task completion. We hypothesized that goal decay in mildEM impairment is reflected by imprecision in task performance at the end of everyday tasks.

**Method:**

54 participants (M age = 73; 39 healthy control [HC], 15 MCI/mild dementia) were videorecorded while preparing a breakfast and a lunch according to specific instructions (Naturalistic Action Task). Performance errors were classified by two coders and another organized them into segments occurring in the beginning, middle or end of the task, all blind to participant diagnosis and study hypothesis. Analyses compared errors in each segment between those with mildEM impairment (MCI/mild dementia) and HC. Correlation analyses examined relations between errors and demographically adjusted standard scores on tests of episodic memory (HVLT and BVMT‐R delayed free recall) and executive function (Digits Backward, Trail Making Test B).

**Result:**

Participants with mildEM impairment made significantly more errors than healthy controls only during the end task segment, t(52) = 2.83, p = .04. The groups did not differ in total errors during the beginning (p = .35) or middle task segments (p = .08). Correlations including the full sample showed significant relations between total errors during the end segment and scores on tests of episodic memory (HVLT r = ‐.33, p = .01; BVMT r = ‐.37, p <.01) but not scores on tests of executive function (Digits Backward r = .01, p = .92; TMT‐B r = .01, p = .97).

**Conclusion:**

Consistent with hypotheses informed by the Goal‐Control Model, older adults with mildEM impairment exhibited increased imprecision towards the end of the task, suggesting that even those with mildEM deficits can experience premature decay of task goals. Behavioral strategies to strengthen goal activations, including reminder cues and/or overt verbalization of task goals, should be investigated as an avenue to improve everyday function in older adults with mild memory difficulties.

